# Integrative taxonomy, distribution, and host associations of *Geocenamus brevidens* and *Quinisulcius capitatus* from southern Alberta, Canada

**DOI:** 10.21307/jofnem-2021-015

**Published:** 2021-03-01

**Authors:** Maria Munawar, Dmytro P. Yevtushenko, Pablo Castillo

**Affiliations:** 1 Department of Biological Sciences, University of Lethbridge, 4401 University Drive W, Lethbridge, AB, T1K 3M4, Canada; 2 Institute for Sustainable Agriculture (IAS), Spanish National Research Council (CSIC), Campus de Excelencia Internacional Agroalimentario, ceiA3, Avenida Menéndez Pidal s/n, 14004 Córdoba, Spain

**Keywords:** *Geocenamus brevidens*, *Quinisulcius capitatus*, Stunt nematodes, Integrative taxonomy, Phylogeny, Morphology, New record

## Abstract

Two stunt nematode species, *Geocenamus brevidens* and *Quinisulcius capitatus*, were recovered from the potato growing regions of southern Alberta, described and characterized based on integrative taxonomy. Morphometrics, distribution, and host associations of both species are discussed. The Canadian populations of both species displayed minor variations in morphometrical characteristics (viz., slightly longer bodies and tails) from the original descriptions. The populations of *G. brevidens* and *Q. capitatus* species examined in this study are proposed as standard and reference populations for each respective species until topotype specimens become available and molecularly characterized. Phylogenetic analyses, based on partial 18S, 28S, and ITS sequences, placed both species with related stunt nematode species. The present study updates the taxonomic records of *G. brevidens* and *Q. capitatus* from a new location, southern Alberta, Canada, and will aid in the decision whether these stunt nematodes should be included in nematode management programs.

The soil, climate, and well-developed irrigation system in the southern region of the province make Alberta one of the most productive places in Canada to grow potatoes, with 20.4% of the country’s total yield reported in 2019 (Statistics Canada, 2020). Alberta also hosts the world’s leading potato processors. Planted areas and crop yields in this province have been increasing steadily to meet the growing demand for potato products. However, disease incidence remains a major limiting factor in profitable potato production. Among the major potato diseases, the potato early dying (PED) complex results in premature plant senescence and can decrease potato marketable yield by as much as 50% (Row and Powelson, 2002). The root-lesion nematode *Pratylenchus penetrans* is a known contributor to PED, along with the fungal wilt pathogen *Verticillium dahliae* and to a lesser extent *V. albo-atrum*. The possible role of other plant-parasitic nematodes in PED development is unknown, although several studies noted the co-occurrence of stunt nematodes and root-lesion nematodes (Smiley et al., 2004; Thompson et al., 2008).

Stunt nematodes are ectoparasites, polyphagous in nature and commonly found in vegetable fields, grasslands, and forest soils. The plant damage caused by these nematodes is difficult to detect; indeed, the impact is a challenge to ascertain as these nematodes either feed directly or potentiate the disease complexes formed by other plant pathogens (Singh et al., 2013). Previous studies detected the presence of 15 stunt nematode species from Canada (Geraert, 2011); however, the recent records only indicate the genus level identification (Pereira, 2018; Wallace, 2016), revealing a gap in our understanding of stunt nematodes inhabiting Canadian soils.

In the present study, two stunt nematode species belonging to the genera *Geocenamus* and *Quinisulcius* were detected in the potato growing regions of southern Alberta, with the latter species being the first record in Canada. Both species were examined morphologically and identified as *Geocenamus brevidens* and *Quinisulcius capitatus*. Because both of these are considered plant-parasitic species in other countries (Smiley et al., 2006; Thompson et al., 2008), the aim of the present study was to (i) provide a detailed molecular and morphometric characterization of both species, (ii) provide extensive information on the species distribution and host associations, and (iii) study the phylogenetic relationship of *G. brevidens* and *Q. capitatus* with other stunt nematode species. The results of this study will lay the foundation for assessing the damage potential of these species on potato production so as to benefit growers and researchers involved in nematode management programs.

## Materials and methods

### Nematode isolation and morphological studies

Nematodes were isolated from soil samples using the modified Cobb sieving and flotation-centrifugation method (Jenkins, 1964). For morphometric studies, nematodes were killed and fixed in hot formalin (4% formaldehyde), processed by ethanol-glycerin dehydration, as described by Seinhorst (1959) and modified by De Grisse (1969), and mounted on permanent slides. Measurements of the mounted specimens were taken using light micrographs prepared on a Zeiss Axioskope 40 microscope equipped with a Zeiss Axiocam 208 camera (Carl Zeiss Microscopy, Jena, Germany).

### DNA extraction, PCR amplification, and sequencing

DNA samples were prepared from nematodes according to Maria et al. (2018). Three sets of DNA primers (Integrated DNA Technologies, Coralville, IA, USA) were used in the PCR analyses to amplify nucleotide sequences of the partial 18S, 28S (LSU), and ITS of ribosomal RNA genes (rDNA). The partial 18S region was amplified with 1813F and 2646R primers (Holterman et al., 2006). The LSU rDNA regions were amplified using 28–81for and 28–1006rev primers (Holterman et al., 2008), and the ITS was amplified with the F194 (Ferris et al., 1993) and AB28-R primers (Curran et al., 1994). PCR conditions were as described by Holterman et al. (2006, 2008) and Ferris et al. (1993). PCR products were resolved in 1% agarose gels and visualized by staining with GelRed (Biotium, Fremont, CA, USA). Amplified DNA fragments were purified following the manufacturer’s protocol (Omega Biotek, Norcross, GA, USA), ligated into the pJET1.2 vector (Thermo Fisher Scientific, Mississauga, ON, Canada), and introduced into *Escherichia coli* DH5α-competent cells (Thermo Fisher Scientific). The presence of the insert-containing plasmids in transformed *E. coli* cells was confirmed by PCR. Plasmid DNA was isolated and purified according to the manufacturer’s instructions (Omega Biotek). The DNA inserts were sequenced at Genewiz, Inc (South Plainfield, NJ, USA) using primers matching the flanking vector sequence.

### Phylogenetic analyses

Sequenced genetic markers from the nematodes examined in the present study (after discarding primer sequences and ambiguously aligned regions), along with several stunt nematode sequences obtained from the GenBank database, were used in the phylogenetic reconstruction. Outgroup taxa for each dataset were selected based on previously published studies (Handoo et al., 2014; Maria et al., 2020; Nguyen et al., 2019). Multiple-sequence alignments of the newly obtained and published sequences were made using the FFT-NS-2 algorithm of MAFFT V.7.450 (Katoh et al., 2019). Sequence alignments were visualized with BioEdit (Hall, 1999) and manually edited by Gblocks ver. 0.91b (Castresana, 2000) in the Castresana Laboratory server (http://molevol.cmima.csic.es/castresana/Gblocks_server.html) using options for a less stringent selection (minimum number of sequences for a conserved or a flanking position: 50% of the number of sequences +1; maximum number of contiguous nonconserved positions: 8; minimum length of a block: 5; allowed gap positions: with half).

Phylogenetic analyses of the sequence datasets were conducted based on Bayesian inference (BI) using MRBAYES 3.2.7a (Ronquist and Huelsenbeck, 2003). The best-fit model of DNA evolution was calculated with the Akaike information (AIC) of JMODELTEST V.2.1.7 (Darriba et al., 2012). The best-fit model, the base frequency, the proportion of invariable sites, substitution rates, and the gamma distribution shape parameters in the AIC were used for phylogenetic analyses. BI analyses were performed under a general time-reversible model, with a proportion of invariable sites and a rate of variation across sites (GTR + I + G) for the partial 18S, 28S, and ITS rRNA regions. These BI analyses were run separately per dataset with four chains for 2 × 10^6^ generations. The Markov chains were sampled at intervals of 100 generations. Two runs were conducted for each analysis. After discarding burn-in samples of 10% and evaluating convergence, the remaining samples were retained for more in-depth analyses. The topologies were used to generate a 50% majority-rule consensus tree. Posterior probabilities (PP) are given on appropriate clades. Trees from all analyses were edited using FigTree software V.1.4.4 (http://tree.bio.ed.ac.uk/software/figtree/).

## Results

### Systematics

**Figure 1:**
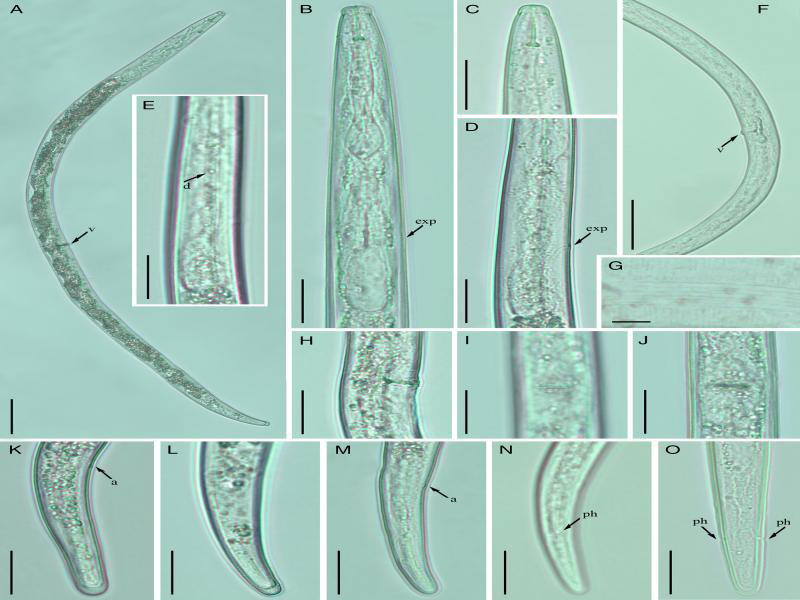
Light photomicrographs of *Geocenamus brevidens.* (A) Entire female, (B) Esophageal region, (C) Lip region, (D) Posterior esophageal region, (E) Deirids, (F) Posterior region with complete reproductive system, (G) Lateral lines, (H-J) Vulval region, (K-O) Female tails. Scale bars: (A) 50 μm; (B-D, E, H-O) 20 μm, (F) 50 μm, (G) 5 μm. Arrows point to (a) anus, (d) deirids, (exp) excretory pore, (ph) phamsid, and (v) vulva.

*Geocenamus brevidens* (Allen, 1955) Siddiqi, 1970 (Fig. 1 and Table 1).

**Table 1.** *Geocenamus brevidens* female morphometrics.

Characters	Present study		Allen (1955) ^a^	Siddiqi (1961)	Alvani et al. (2017)	Tzortzakakis et al. (2018)
Origin	Canada		USA	India	Iran	Greece
Host	Potato field	Grass	Grass	Cauliflower, cabbage, mint, potato, pea	Jujube, saffron, barberry	Cultivated olives
*n*	15	10	11	15	8	3
Body length	667.8 ± 64.2 (590.9-811.0)	687.1 ± 53.3 (604.0-752.0)	540-690	550-850	650 (600-718.5)	564 (490-698)
a	35.4 ± 2.8 (25.5-38.5)	28.2 ± 1.8 (24.8-31.1)	23-27	22-29	26.5 (23.9-29)	26.3 (22.9-30.3)
b	5.0 ± 0.4 (4.6-5.9)	4.9 ± 0.3 (4.5-5.6)	4.2-5.2	4.6-6.0	5.0 (4.5-5.2)	4.4 (4.0-5.1)
c	12.7 ± 0.6 (11.7-13.6)	14.3 ± 1.1 (12.7-15.9)	11-13	12-17	13.7 (13.7-15.7)	14.3 (12.9-17.0)
c'	4.2 ± 0.3 (3.8-4.9)	3.2 ± 0.2 (3.0-3.5)	2.5-4.4	–	2.7 (2.4-3.2)	3.0 (2.9-3.2)
V	56.6 ± 1.6 (53.8-61.0)	56.2 ± 1.2 (54.5-58.1)	52-58	54-61	55.6 (54.5-57)	54.7 (52-58)
MB	48.6 ± 2.0 (46.2-54.3)	51.1 ± 1.5 (49.6-53.7)	42-47	–	(41.1-46.5)	–
Lip height	3.6 ± 0.3 (3.1-4.0)	3.6 ± 0.3 (3.0-4.0)	–	–	–	–
Lip width	7.2 ± 0.5 (6.1-7.7)	8.0 ± 0.2 (7.5-8.3)	6.5–8.5	–	–	–
Stylet length	16.2 ± 0.7 (15.0-17.5)	17.0 ± 0.5 (16.0-17.6)	14-16	13-15	16.3 (16-16.5)	14.3 (13-16)
Median bulb length	14.2 ± 1.3 (12.3-16.4)	15.1 ± 0.6 (14.3-15.8)	13-16	–	–	–
Median bulb width	9.9 ± 0.9 (8.5-11.4)	10.3 ± 0.9 (9.2-11.7)	9-12	–	–	–
Anterior end to excretory pore	103.3 ± 6.7 (90.3-112.5)	106.1 ± 1.9 (103.0-109.0)	–	–	–	–
Pharynx length	133.5 ± 6.9 (120.3-144.0)	139.6 ± 5.8 (130.0-148.0)	110-118	–	129.9 (118.5-141)	127.7 (123-136)
Maxim body width	18.6 ± 1.7 (15.5-21.2)	24.4 ± 1.2 (22.3-26.2)	19-24	–	100.2 (90.5-110)	21.3 (19.0-23.0)
Vulva body length	19.4 ± 1.9 (16.4-22.3)	23.5 ± 1.3 (21.0-25.3)	–	–	24.7 (21.5-30)	–
Anal body width	12.6 ± 1.0 (10.3-14.2)	14.9 ± 0.5 (13.7-15.3)	–	–	17.1 (15.5-19)	13.0 (12.0-14.0)
Tail length	52.5 ± 3.7 (46.0-59.8)	48.2 ± 2.2 (46.0-52.0)	34-58	–	47.5 (42-53)	39.3 (38-41.0)
Phasmid position	Posterior to middle of tail	Posterior to middle of tail	Posterior to middle of tail	–	–	–

**Notes:** All measurements are in µm and in the form: mean ± standard deviation (range). ^a^Original description.

### Description

#### Female

Body straight with curved tail region or open C-shaped. The cuticle annulated, lateral field with six incisures. Cephalic region continuous, broadly rounded with 3 to 4 indistinct annuli, basal ring of head framework shallow, distinctively arched. Stylet 15 to 17 µm long with rounded basal knobs. Dorsal gland opening (DGO) 1.5 to 3.0 µm posterior to basal knobs. Median bulb spherical to oval, well-developed, central valve plates bean-shaped slightly anteriorly. Isthmus slender encircled with nerve ring. Deirids were present (observed in few specimens). Excretory pore anterior to the basal esophageal bulb. Hemizonid 2 to 3 body annuli long situated slightly anterior to the excretory pore. Cardia rounded, intestine densely globular. Ovaries outstretched, vulva with small epiptygma, which mostly appear as protruding lips, vagina inclined anteriorly covering half of the corresponding body diameter. Spermatheca rounded, scarcely filled with sperm. Tail subcylindrical, gradually tapering to a smooth broadly rounded or truncated terminus. Hyaline region of tail prominent 3.2–5.5 µm long. Phasmids near or slightly posterior to mid-tail.

#### Male

Not found.

#### Remarks

*Geocenamus brevidens (=Tylenchorhynchus brevidens*, Allen, 1955 and *Merlinius brevidens*, Siddiqi, 1970) was originally described in the rhizosphere of grass from the USA by Allen in 1955. Since then, this species has been reported from diverse climate regions and agricultural environments (Table 3). Despite its wide distribution, few morphometrical studies are available for comparison (Table 1). *Geocenamus brevidens* was also reported from potato fields in Ontario (Olthof et al., 1982), although no morphological and morphometric studies were presented. Hence, we consider our population of *G. brevidens* as a Canadian population. We observed that nematodes of the Canadian population of *G. brevidens* were slightly longer and wider than in the original and other reported descriptions, with the exception of those from India (Siddiqi, 1961). The Canadian population morphometrical values were in good agreement with the Indian population, except for the stylet length, which was longer in the Canadian population, 15.0 to 17.5 vs 13.0 to 15.0 µm. The other morphological characteristics, i.e., lip and tail morphology, overall body habitus, and vulva appearance, were consistent with the original description. Males were described in the original description and by Siddiqi (1961), but in later reports, no males were ever detected. Geraert (2011) mentioned that males were uncommon in *G. brevidens*; the Canadian population was also found devoid of males. In the original and subsequent descriptions, the authors did not observe the presence of spermatheca in *G. brevidens* (Allen, 1955; Alvani et al., 2017; Tzortzakakis et al., 2018). On the other hand, round-shaped spermathecae were found in the Canadian and Indian populations. The other characteristics noted by Siddiqi (1961) in the Indian population were tightly closed stylet knobs, indistinct anus, and rather cylindrical tail. In the Canadian population, the anus was prominent, stylet knobs were rounded, and the tail was cylindrical with a broadly rounded, somewhat truncated terminus. We consider these small differences between *G. brevidens* populations to be due to intraspecific geographic variation.

**Table 2.** *Quinisulcius capitatus* female morphometrics.

Characters	Present study	Allen (1955)^a^	Hopper (1959)	Loof (1959)	Siddiqi (1961)	Knobloch and Laughlin (1973)	Maqbool (1982)	Vovlas (1983)	Mekete et al. (2008)
Origin	Canada	USA	USA	Italy	India	Mexico	Pakistan	Italy	Ethiopia
Host	Grass	Pear	okra	Date palm	Grass Lily	Grass, pasture grass, wild poppy, barrel cactus, cotton	Potato	Maize	Coffee
*n*	20	13	5	14	8	–	5	25	10
Body length	810.3 ± 44.6 (744.0-911.0)	630-850	650-710	631-830	700-810	520-630	610-730	641-730	630-790
*a*	41.4 ± 1.8 (38.6-43.7)	30-38	30.2-36.2	31-37.6	30-35	29-33	30-33	31-35	30.9-38.6
*b*	5.5 ± 0.3 (5.0-6.3)	5.0-5.8	4.6-4.9	4.7-5.5	4.5-5.5	4.4-5.3	6.8-7.4	4.1-5.3	–
*c*	22.4 ± 1.1 (19.9-23.8)	12-17	14.5-16.1	14.3-17.4	15-17	15-17	3.8-4.9	13-16	15.3-17.6
*c′*	2.6 ± 0.2 (2.2-3.2)	–	–	–	–	–	–	2.8-3.2	
*V*	57.4 ± 1.5 (53.4-59.8)	51-58	55.2-56.9	53.5-58	54-56.5	52-59	53-56	54-57	54.7-63.6
MB	52.3 ± 1.7 (47.4-56.1)	–	–	–	–	–	–	57-60	–
Lip height	4.0 ± 0.2 (3.7-4.4)	–	–	–	–	–	–	–	–
Lip width	7.6 ± 0.4 (6.9-8.3)	–	–	–	–	–	–	–	–
Stylet length	18.3 ± 1.0 (15.5-20.4)	16-18	16.5-17.3	16-18	16-17	16-17	18.4-19.5	15-17	15-18
Median bulb length	14.0 ± 1.6 (11.3-16.9)	–	–	–	–	–	–	–	–
Median bulb width	10.4 ± 1.4 (8.4-14.2)	–	–	–	–	–	–	–	–
Anterior end to excretory pore	128.6 ± 5.3 (121.0-139.0)	–	–	–	–	–	–	119-128	121
Pharynx length	147.8 ± 5.8 (140.2-159.0)	–	–	–	–	–	–	–	–
Maxim body width	19.0 ± 1.5 (16.9-21.3)	–	–	–	–	–	–	–	–
Vulva body length	18.4 ± 1.3 (15.7-20.8)	–	–	–	–	–	–	–	–
Anal body width	13.8 ± 1.1 (11.2-15.2)	–	–	–	–	–	–	–	–
Tail length	35.8 ± 2.4 (31.3-40.4)	–	–	–	–	–	–	40-48	–
Phasmid position	Middle of tail	Middle of tail	Middle of tail	Anterior to middle of tail	Anterior to middle of tail	Anterior to middle of tail	Anterior to middle of tail	Anterior to middle of tail	–

**Notes:** All measurements are in µm and in the form: mean ± standard deviation (range). ^a^Original description.

**Table 3.** Worldwide distribution and host plant association of *Geocenamus brevidens* and *Quinisulcius capitatus*.

No.	Country	Host associations	References
*G. brevidens*			
		Americas	
1	USA	WheatPotato	Mayol (1981), Smiley et al. (2006) Olthof et al. (1982)
		Wheat grass	Griffin and Asay (1996)
		Pasture filed, vegetable and horticultural crops	Hafez et al. (2010)
		Asia	
2	Iran	Wheat	Ghaderi et al. (2014)
		Jujube, saffron, barberry	Alvani et al. (2017)
		Africa	
3	South Africa	Wheat	Jordaan et al. (1992)
4	Egypt	Soybean	Salem et al. (1994)
		Europe	
5	Spain	Cereals, sunflower, wheat	Tobar et al. (1995a, 1995b)
		Chickpea	Castillo et al. (1996)
		Grasslands	Talavera and Navas (2002)
		Olives	Palomares-Rius et al. (2015)
6	Slovakia	Hop gardens	Lišková and Rencˇo (2007)
7	Czech Republic	Hop gardens	Cˇermák et al. (2011)
8	Turkey	Cultivated plants	Kasapog˘lu et al. (2014)
		Cotton, barley, melons, tobacco, Watermelons, wheat, lentils	Kasapog˘lu Uludamar et al. (2018)
9	Greece	Olives	Tzortzakakis et al. (2018)
10	Poland	Jerusalem artichoke	Zapałowska and Skwiercz (2018)
		Oceania	
11	Australia	Cereal fields	Meagher (1970)
		Wheat	Thompson et al. (2008, 2010)
		Millet, soybean, grasses	Owen et al. (2014)
*Q. capitatus*			
		Americas	
1	Ecuador	Avocado, barley, bean, carrot, cucumber, lettuce, pea, onion, tomato, soybean, sugarcane	Bridge (1976)
2	Argentina	Corn, sunflower	Doucet (1986)
3	USA	Okra	Hopper (1959)
		Wild poppy, barrel cactus, cotton	Knobloch and Laughlin (1973)
		Tobacco	Ponchillia (1975)
		Red clover, Kentucky bluegrass	Malek (1980)
		Sorghum	Cuarezma-Teran and Trevathan (1985)
		Potato	Hafez et al. (2010)
		Switchgrass	Cassida et al. (2005)
		Asia	
4	Pakistan	Potato	Maqbool (1982)
			Maqbool and Hashmi (1986)
5	India	Lily	Siddiqi (1961)
		Potato	Krishna Prasad (2008)
6	Iran	Cultivated crops	Kheiri et al. (2002)
		Africa	
7	Ethiopia	Coffee	Mekete et al. (2008)
8	South Africa	Potato	Marais et al. (2015)
		Soybean	Mbatyoti et al. (2020)
		Europe	
9	Italy	Lily, maize	Loof (1959), Vovlas (1983)
10	Bulgaria	Apple	Braasch (1978)
11	Cypress	Grapes	Antoniou (1981)
12	Turkey	Tomato, tobacco	Kasapog˘lu Uludamar et al. (2018)
		Oceania	
13	New Zealand	Tomato, tobacco, squash	Knight et al. (1997)

#### Habitat and locality

Two *G. brevidens* populations were found in the present study. The first population was found in a potato field (latitude 50° 34′ 43″ N; longitude – 112° 30′ 34.7″ W) of Vulcan county, whereas the second one was discovered in the rhizosphere of grass growing on the headland (uncultivated field margin) (latitude 49° 47′ 22.66″ N; longitude – 112° 13′ 23″ W) of Taber Rural Municipality, Alberta, Canada.

**Figure 2:**
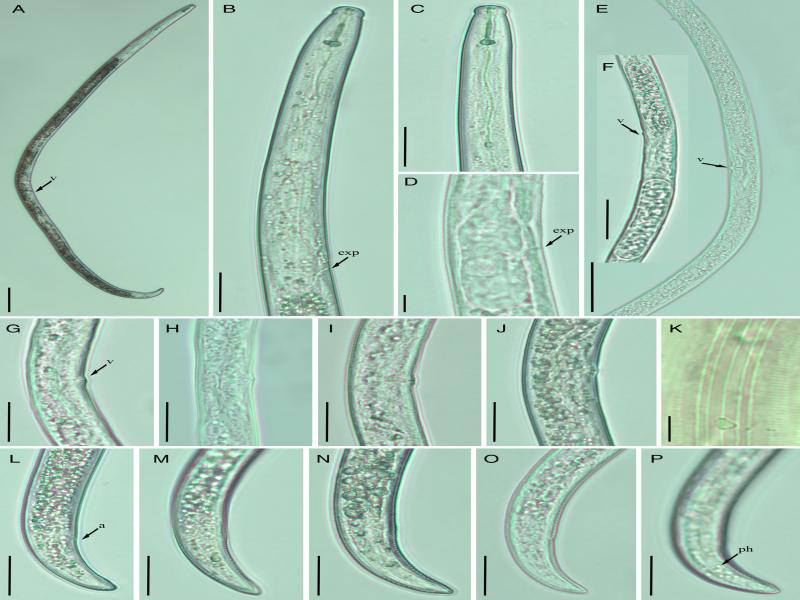
Light photomicrographs *Quinisulcius capitatus.* (A) Entire female, (B) Esophageal region, (C) Lip region, (D) Basal esophageal bulb, (E) Posterior region with complete reproductive system, (F) Posterior region with eggs, (G-J) Vulval region, (K) Lateral lines, (L-P) Female tails. Scale bars: (A) 100 μm, (B, C, F-J; L-P) 20 μm, (E) 50 μm, (D, K) 5 μm. Arrows point to (a) anus, (d) deirids, (exp) excretory pore, (ph) phasmid, and (v) vulva.

Quinisulcius capitatus (Allen, 1955) Siddiqi, 1971 (Fig. 2 and Table 2).

### Description

#### Female

Body open C-shaped, appeared concave at vulval level. Cuticle annulated, lateral field with five incisures. Cephalic region continuous with slight depression at the junction of lip and body, broadly rounded with a few indistinct lip annuli. Stylet robust, 15 to 20 µm long with rounded basal knobs. DGO 2.5 to 4.0 µm posterior to basal knobs. Median bulb rounded, well developed with conspicuous central valve plates. Isthmus slender, encircled with nerve ring. Deirids not seen. Excretory pore at the middle of basal esophageal bulb. Hemizonid inconspicuous, 2 to 3 body annuli long, situated 3 to 4 annuli anterior to excretory pore. Cardia rounded, intestine densely globular. Ovaries outstretched, vulva with protruding lips, vagina straight covering more than half of the corresponding body diameter. Spermatheca weakly developed, rounded. Tail conoid, distinctly annulated, gradually tapering to a heart or V-shaped terminus. Phasmids near or slightly posterior to mid-tail.

#### Male

Not found.

#### Remarks

*Quinisulcius capitatus (=*Tylenchorhynchus capitatus* (Allen, 1955)* Siddiqi, 1971) was originally described in the rhizosphere of pear from the USA by Allen in 1955. Afterward, this species has been found in various geographic and agricultural locations (Table 3). In the present study, the nematodes of the Canadian population of *Q. capitatus* were slightly longer and wider than those described in the original and other reports. Because of the longer body, the Canadian population had a longer stylet and tail, whereas the other morphological characteristics, such as lip and tail morphology, indistinct hemizonid, weakly developed spermatheca and vulva appearance, corresponded well with the original description. Male (*n* = 4) was only described in Allen (1955); however, no males were detected in any other reported population (Hopper, 1959; Knobloch and Laughlin, 1973; Maqbool, 1982; Mekete et al., 2008; Siddiqi, 1961). Similarly, no male was found in the Canadian population. Of 17 valid species of *Quinisulcius*, males were not described for 10 species (Geraert, 2011). We speculate that males of *Quinisulcius* are either very rare or do not have a significant role in reproduction. Another species, *Q. acti*, was described from the rhizosphere of okra by Hopper in 1959; however, this species was soon synonymized with *Q. capitatus* (Siddiqi, 1961). Knobloch and Laughlin (1973) emphasized the number of tail annuli and the lateral line incisures characteristics at the phasmid level to reinstate the species *Q. acti*. However, this action was not accepted, and the species was regarded as synonym of *Q. capitatus* (Geraert, 2011). Several more species, namely *Q. nilgiriensis* (Seshadri et al., 1967), *Q. himalayae* (Mahajan, 1974), *Q. solani* (Maqbool, 1982), *Q. paracti* (Ray and Das, 1983), and *Tylenchorhynchus maqbooli* (Mizukubo et al., 1993), were described as new members of the *Quinisulcius* genus. Close scrutiny of these descriptions indicates that these species do not manifest any significant differences from *Q. capitatus*. The only difference mentioned by the authors was the varying number of lip and tail annuli. In the present study, we noted that the number of annuli on the lip or tail region of *Q. capitatus* are variable and cannot be considered valid characters for species differentiation. In the majority of our specimens, lip annuli were not clearly visible and tail annuli were difficult to count. In fact, most of the tail annuli became faint near the terminus, thereby posing a challenge to accurately count their number. Similarly, the lateral incisures at the level of or past the phasmid were not a constant character. Therefore, we suggest using the robust morphological characters for species differentiation.

#### Habitat and locality

This population was found in the rhizosphere of grass growing on the headland of a planted wheat field that had been in a crop rotation cycle with potatoes (latitude 49° 52′ 37.4″ N; longitude – 111° 56′ 37.5″ W); Taber Rural Municipality, Alberta, Canada.

## Molecular characterization and phylogeny

The sequences of partial 18S (GenBank accession numbers MW029450, MW029451 for *G. brevidens*; MW023248, MW023249 for *Q. capitatus*), 28S (MW029449 for *G. brevidens*, MW023387 for *Q. capitatus*), and the ITS region of the rRNA (MW029446, MW029447, MW029448 for *G. brevidens*; MW027537, MW027538 for *Q. capitatus*) of both species were obtained.

**Figure 3:**
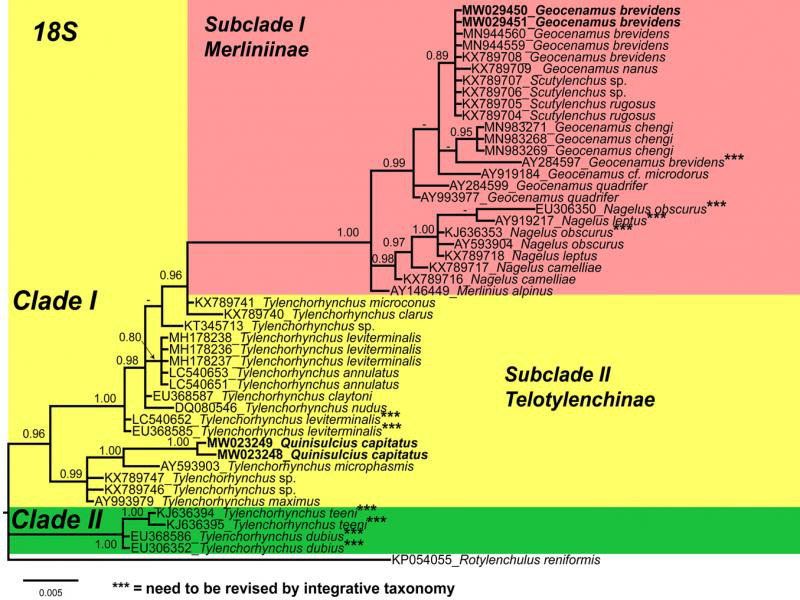
Phylogenetic relationships within selected genera of subfamily Telotylenchinae and subfamily Merliniinae as inferred from Bayesian analysis using the 18S of the rRNA gene sequence dataset with the GTR + I + G model (lnL = 1,910.5101; AIC = 4,017.0201; freq A = 0.2500; freq C = 0.2500; freq G = 0.2500; freq T = 0.2500; R(a) = 1.0000; R(b) = 3.9248; R(c) = 1.0000; R(d) = 1.0000; R(e) = 4.6930; R(f) = 1.0000). Posterior probability of more than 70% is given for appropriate clades. Newly obtained sequences are indicated in bold. *** need to be revised by integrative taxonomy.

**Figure 4:**
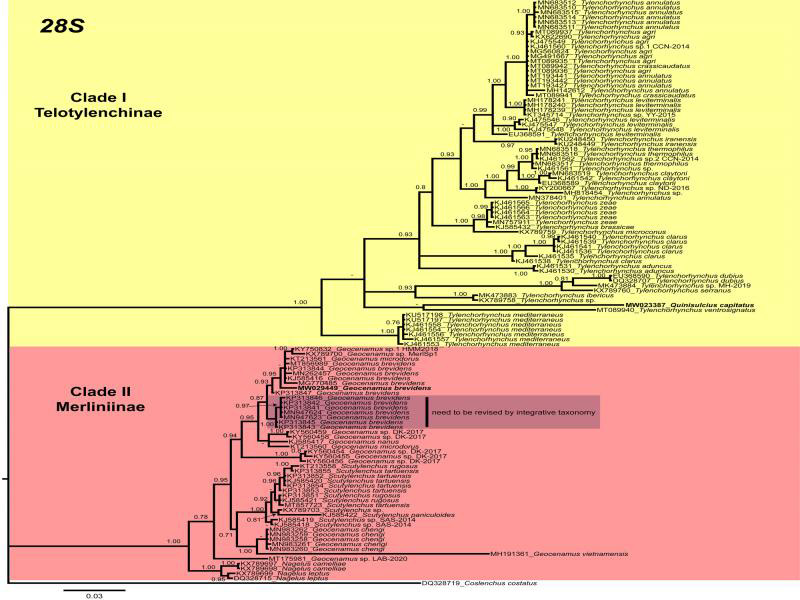
Phylogenetic relationships within selected genera of subfamily Telotylenchinae and subfamily Merliniinae as inferred from Bayesian analysis using the 28S of the rRNA gene sequence dataset with the GTR + I + G model (lnL = 6,015.1425; AIC = 12,526.2851; freq A = 0.1987; freq C = 0.2072; freq G = 0.3206; freq T = 0.2736; R(a) = 0.4322; R(b) = 2.5823; R(c) = 1.2662; R(d) = 0.2497; R(e) = 5.4146; R(f) = 1.0000). Posterior probability of more than 70% is given for appropriate clades. Newly obtained sequences are indicated in bold.

**Figure 5:**
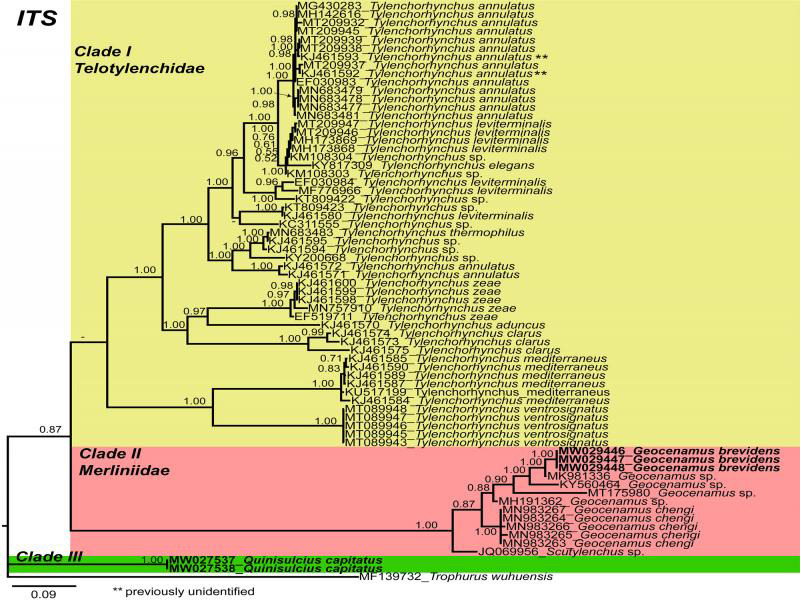
Phylogenetic relationships within selected genera of subfamily Telotylenchinae and subfamily Merliniinae as inferred from Bayesian analysis using the ITS of the rRNA gene sequence dataset with the GTR + I + G model (lnL = 10,413.7629; AIC = 21119.5049; freq A = 0.1932; freq C = 0.2202; freq G = 0.2725; freq T = 0.3141; R(a) = 0.8338; R(b) = 3.3701; R(c) = 1.6297; R(d) = 0.6490; R(e) = 3.3701; R(f) = 1.0000). Posterior probability of more than 70% is given for appropriate clades. Newly obtained sequences are indicated in bold. **previously unidentified.

Phylogenetic relationships among the isolates were determined separately for each dataset using Bayesian inference (BI) (Figs. 3-5). The 18S tree presents two distinct main clades (Fig. 3), Clade I is well supported (PP=0.86) and further divided into two subclades. The subclade (I) consists of subfamily Merliniinae and subclade (II) represents subfamily Telotylenchinae. The Canadian population of *G. brevidens* grouped with other *G. brevidens* populations from GenBank in subclade (I). However, it is noted that another population of *G. brevidens* (AY284597) from the Netherlands distantly arranged from the *G. brevidens* clade. Additionally, sequences of *Nagelus obscurus* (AY593904, KJ636353, and EU306350), from the Netherlands and Belgium do not cluster together. Therefore, these data suggest that a misidentification is probable and requires a detailed re-evaluation based on integrative taxonomy for ascertaining their species status. In subclade (II), the *Q. capitatus* grouped with *Tylenchorhynchus microphasmis* (AY593903), *T. maximus*, and two unidentified *Tylenchorhynchus* spp. Since the *Quinisulcius* sequences deposited in the GenBank were the first sequences deposited for this genus, we anticipate that inclusion of more *Quinisulcius* sequences will rearrange the position of *Q. capitatus*. We noted that the sequences of *T. leviterminalis* (LC540652, EU368585) arranged distantly from the *T. leviterminalis* clade. Handoo et al. (2014) suggested that *T. leviterminalis* may consist of a species complex; therefore, a detailed re-evaluation based on integrative taxonomy is required to determine the exact status of these populations.

The 28S tree presents two well-supported (PP = 1.00) main clades (I and II; Fig. 4), including members of the Telotylenchinae (I) subfamily, while clade (II) was formed of subfamily Merliniinae. Our phylogenetic analyses place *Q. capitatus* within the species of *Tylenchorhynchus* in Clade (I). However, due to a lack of *Quinisulcius* sequences, phylogenetically close species cannot be determined. Clade (II) indicates that the Canadian population of *G. brevidens* grouped with *G. brevidens* populations from China (MT856989, unpublished), Iran (KP313844, Alvani et al., 2017), South Africa (MN262457, unpublished), Iran (KJ585416, Ghaderi et al., 2014), and Greece (MG770485, Tzortzakakis et al., 2018). However, a few additional populations of *G. brevidens* from Iran (KP313841, KP313842, KP313843, KP313845, KP313846, Alvani et al., 2017; MN947623, MN947623, unpublished) formed another subclade. The morphological and morphometric information of these last populations were not provided by the authors, which indicates that these populations likely require re-evaluation based on detailed integrative taxonomy. Finally, the ITS tree presents three distinct major clades (Fig. 5). Clade (I) is poorly supported (PP = 0.64) and consists of *Tylenchorhynchus* species. Very few *Geocenamus* species have been molecularly characterized; the Canadian population of *G. brevidens* grouped with *G. chengi* and unidentified populations of *Geocenamus* species in clade (II). It is noted that in this tree, *Q. capitatus* does not cluster within *Tylenchorhynchus* species but independently appeared as a different, well-supported clade (III) (PP = 1.00).

In the present study, we observed that *G. brevidens* is a cosmopolitan species reported from several countries. However, all the *G. brevidens* sequences that have been submitted to GenBank were not supplemented with integrative taxonomic descriptions. Therefore, their true identity is difficult to ascertain. Moreover, molecular information about all the valid species of *Quinisulcius* is necessary in order to establish their phylogenetic relationship within the Telotylenchinae.

## Discussion

Currently, the genus *Geocenamus* contains over 70 species (Maria et al., 2020), with eight of them found in Canada (Geraert, 2011). Among the latter, *G.laminatus* (Wu, 1969) Brzeski, 1991 and *G. longus* (Wu, 1969) Tarjan, 1973 Wu, 1969 were described from Alberta, with *G. brevidens* being the third species discovered in this province. *Geocenamus brevidens* is a native American species described by Allen (1955) in the rhizosphere of grass; however, studies have indicated that it has a high rate of occurrence in European countries, followed by Asia and Australia (Table 3).

The pathogenic nature and soil preference of *G. brevidens* were previously examined by several researchers, who demonstrated that this species requires relatively cooler soils and low temperature for achieving an optimal reproduction rate. Moreover, the damage potential of *G. brevidens* increased in clay sand and sandy loam soils (Griffin, 1994; Griffin and Asay, 1996; Malek, 1980; Mayol, 1981; Smiley et al., 2006). In the present study, we detected high numbers of *G. brevidens* in the grass soil, but relatively low numbers in the farmed field. The lower population density in the farmed field could be related to the amount of disturbance in the soil: the field was under the agricultural practice of tillage and crop rotation, whereas the grass on the headland remained undisturbed.

In the USA, *G. brevidens* causes stunting in small cereal crops (Langdon et al., 1961), chlorotic tillers in wheat (Mayol, 1981), reduced growth in wheatgrass (Griffin and Asay, 1996), and reduced yield in wheat (Smiley et al., 2006). Additionally, *G. brevidens* has been found associated with the root lesion nematodes in cultivated cereal fields of Australia (Owen et al., 2010; Thompson et al., 2008). In European and Asian countries, the presence of *G. brevidens* has been recorded both in agricultural and horticultural fields (Alvani et al., 2017; Castillo et al., 1996; Čermák et al., 2011; Kasapoğlu-Uludamar et al., 2018; Palomares-Rius et al., 2015; Tobar et al., 1995), although no considerable plant damage was associated with this species. Considering the observed damage caused by this species in the USA, we hypothesize that *G. brevidens* has a much higher impact on agricultural crops in its originally described geographic range.

The genus *Quinisulcius* contains over 17 valid species, but none of them was ever reported from Canada (Geraert, 2011). Hence, our study presents the first record of *Q. capitatus* in Canada. This species was originally described from the USA (Allen, 1955) and is widely distributed throughout the American continent, followed by Europe and Africa (Table 3). It has been demonstrated that *Q. capitatus* reproduce well in cooler soils and require low temperature for survival (Malek, 1980). The soil preference of *Q. capitatus* was studied by Maqbool and Hashmi (1986), who found that sandy clay soil is best for achieving a high level of infestation in greenhouse-grown potatoes. *Quinisulcius capitatus* has been reported in association with *Pratylenchus zeae* to cause sorghum root rot and plant growth decline (Cuarezma-Teran and Trevathan, 1985). Moreover, in the presence of *Helicotylenchus dihystera* this *Quinisulcius* species reduced the potato tuber yield up to 14 to 29% (Krishna Prasad and Sharma, 1985). In our study, we found a *Q. capitatus* population in the headland of a farmed field that was previously rotated with potatoes. The discovery of *Q. capitatus* in the vicinity of a cultivated field calls for additional surveys that will likely uncover other populations of *Q. capitatus*.

In this work, populations of *G. brevidens* and *Q. capitatus* species were examined based on morphological, quantitative (morphometrical), and molecular characters. In our 28S phylogenetic trees, *G. brevidens* sequences formed two separated subclades. Our data of *G. brevidens* are coincident with the original descriptions as well as with other populations that have been described using integrative taxonomy (Ghaderi et al., 2014; Tzortzakakis et al., 2018). Consequently, it is imperative to further investigate the populations that formed the second subclade. It is likely that these species belong to other close species of *Geocenamus* rather than to *G. brevidens*. We agree with Handoo et al. (2014) who stated that stunt nematodes present variability in morphological characteristics and overlapping morphometrical values may lead to potential misidentification. Therefore, the only definitive solution for this situation will be to sequence topotype population of *G. brevidens* that may clarify this situation. Moreover, in our phylogenetic trees, *Q. capitatus* grouped with *Tylenchorhynchus* species; the closely related species cannot be determined due to the lack of *Quinisulcius* sequences in GenBank. Based on integrative taxonomical characterization, we propose our *G. brevidens* and *Q. capitatus* as standard and reference populations for each species until topotype specimens become available and molecularly characterized.

The current knowledge of the occurrence and distribution of non-target nematode species in Alberta is very scarce. Here, we provide the descriptions of two stunt nematodes based on an integrative taxonomical approach. Data on geographical distribution and host plant associations of these species are also provided and discussed. The present study will help to update the taxonomic records of *G. brevidens* and *Q. capitatus* from Alberta, Canada. Moreover, our light micrographs and sequence-based information will enable prompt identification of these species. Last, it will be important for future research to conclusively determine whether these stunt nematodes should be incorporated into nematode management programs.
